# Interrater reliability of MRI Neck Imaging Reporting and Data System (NI-RADS) in the follow-up of nasopharyngeal carcinoma after radiation therapy

**DOI:** 10.1007/s11547-025-01982-4

**Published:** 2025-04-01

**Authors:** Andrea Falzone, Marco Parillo, Marinella Neri, Alessandro Marinetti, Matteo Zanini, Francesco Sella, Carlo Cosimo Quattrocchi

**Affiliations:** 1 Radiology, Multizonal Unit of Rovereto and Arco, APSS Provincia Autonoma di Trento, Trento, Italy; 2 Centre for Medical Sciences - CISMed, University of Trento, Trento, Italy

**Keywords:** Head and neck neoplasms, Squamous cell carcinoma, Magnetic resonance imaging, Diagnostic imaging, Reproducibility of results, Practice guideline

## Abstract

**Purpose:**

Evidence supporting the reliability of magnetic resonance imaging (MRI) Neck Imaging Reporting and Data System (NI-RADS) is currently limited. This study aims to evaluate the interrater agreement of MRI NI-RADS among radiologists with varying levels of expertise in nasopharyngeal carcinoma (NPC) patients.

**Material and methods:**

We designed an observational retrospective study to identify follow-up MRIs in patients who had undergone radiation therapy. Five radiologists (2 head and neck experts, 1 general radiologist, and 2 residents in radiology) scored each MRI using NI-RADS. Kappa (*κ*) and percentage of agreement (POA) were calculated for the ultimate score and for each individual feature of the NI-RADS (primary tumor size, signal on T2-weighted images, contrast enhancement, diffusion restriction, and lymph node size). Agreement was analyzed also separately for the first follow-up MRI and subsequent scans.

**Results:**

Thirty patients were included (a total of 97 MRIs per rater). Interreader agreement between all readers was moderate for NI-RADS (*κ* = 0.41; POA = 81%). The first follow-up showed a low reliability between the head and neck expert radiologist and the two radiology residents for both primary tumor contrast enhancement and size assessment (*κ* = 0.02; POA = 31% and *κ* = 0.17; POA = 38%, respectively), while there was a high level of agreement in the analysis of diffusion-weighted imaging (DWI) (*κ* = 0.79; POA = 96%).

**Conclusion:**

MRI NI-RADS has a moderate interrater agreement in NPC patients after radiation therapy. Educational effort should focus on the assessment and interpretation of primary tumor contrast enhancement and size in the first examination performed after treatment, by also considering information derived from DWI.

**Supplementary Information:**

The online version contains supplementary material available at 10.1007/s11547-025-01982-4.

## Introduction

Nasopharyngeal carcinoma (NPC) is a relatively uncommon cancer in regions outside of endemic areas (e.g., East and Southeast Asia) [1]. In Europe, an estimated 5000 new cases were diagnosed in 2020, while Italy reported fewer than 700 new NPC cases in the same year [2]. Radiation therapy is the cornerstone treatment for NPC and is indispensable for achieving a cure in patients with localized disease [1]. Re-irradiation is a tailored treatment option for recurrent NPC, suitable only for carefully chosen patients and administered in specialized centers equipped with dedicated technology [3, 4].

Magnetic resonance imaging (MRI) plays a key role in managing patients with NPC, excelling in delineating the primary tumor and nodal involvement. In fact, it is the most accurate way of defining local and nodal tumor staging. While MRI and positron emission tomography (PET) demonstrate comparable sensitivity in post-treatment surveillance, PET's superior specificity for differentiating post-radiation changes from recurrent disease is often counterbalanced by cost and accessibility limitations. Initial imaging is recommended 3 months post-treatment, followed by regular MRI examinations of the nasopharynx and skull base every 6 months for the first 3 years [1].

Inconsistent reporting practices currently hinder the clarity and understanding of imaging findings in patients with treated head and neck cancer. To address this issue, the American College of Radiology introduced the Neck Imaging Reporting and Data System (NI-RADS) [5, 6]. Originally designed for post-treatment surveillance using contrast-enhanced computed tomography (CT) with or without PET, the NI-RADS has recently been adapted for MRI [7, 8]. The standardized reporting lexicon aids radiologists in navigating the complex post-treatment imaging landscape, often characterized by anatomical alterations from reconstructive surgeries and radiation-induced tissue modifications. Furthermore, NI-RADS facilitates communication with surgeons and supports informed decision-making regarding subsequent patient management [9].

Similar to other RADS [10–12], the MRI NI-RADS requires rigorous validation through interobserver agreement studies. Currently, evidence supporting the reliability of MRI NI-RADS among radiologists, particularly in the NPC patient population, remains limited [9, 13, 14]. This study aims to evaluate the interrater agreement of MRI NI-RADS among radiologists with varying levels of expertise in a cohort of NPC patients undergoing surveillance imaging after radiation therapy.

## Material and methods

### Study design and patient selection

Ethical approval for this retrospective observational study was obtained from the ethics committee in accordance with the 2013 Declaration of Helsinki guidelines (identification code: 2024-087ESA). Given the study's retrospective nature and the exclusive use of anonymized, pre-existing data, informed consent was waived.

The study period encompassed the dates between January 1, 2009, and January 31, 2024. Patients were included if they had a histologically verified diagnosis of squamous cell carcinoma of the nasopharynx and underwent at least two, up to four, head and neck MRI examinations after radiation therapy. Exclusion criteria included cases lacking histological confirmation of NPC, patients receiving non-radiation-based treatments, those with MRI scans solely for staging purposes, and individuals monitored with CT or PET-CT scans.

Patient demographics (sex, age), MRI dates, number of post-radiation MRI scans, nasopharyngeal cancer histology, and treatment outcomes (remission or recurrence) were extracted from electronic medical records and the picture archiving and communication system (PACS).

### MRI protocol

Head and neck MRIs were performed on 1.5T devices (Magnetom Aera, Siemens and Optima MR450w, GE) in three different hospitals using an institutional head and neck cancer protocol, which included the following sequences: axial T1-weighted turbo spin-echo, axial T2-weighted turbo spin-echo, axial and coronal fat-suppressed T2-weighted turbo spin-echo, axial diffusion-weighted imaging (DWI) with corresponding apparent diffusion coefficient map, and 3D contrast-enhanced and fat-suppressed T1-weighted gradient echo after intravenous administration of 0.01 mmol/kg of gadoterate meglumine or gadobutrol. DWI data were missing for thirteen MRI examinations, and contrast-enhanced images were missing for two MRI examinations. All available sequences within each evaluated MRI scan were accessible for review by the readers.

### Image interpretation

Five radiologists of varying experience levels participated in the study by providing reports (A: expert head and neck radiologist with 22 years of experience; B: expert head and neck radiologist with 21 years of experience; C: general radiologist with 20 years of experience; D and E: radiology residents at their third year of postgraduate education). To standardize assessment, all readers received introductory materials on the NI-RADS, including illustrative case examples representing each of the four NI-RADS scores.

All included MRI datasets underwent de-identification by removing any patient-specific information and were subsequently randomized. The anonymized data were displayed on a radiology workstation equipped with dual diagnostic monitors and a single administrative monitor using Synapse PACS (Version 7.3.002, Fujifilm). The current MRI study was presented on the left monitor for simultaneous comparison with the preceding study displayed on the right monitor.

Two separate NI-RADS categories were assigned for each imaging study: one for the primary tumor site and another for cervical lymph nodes. The higher of these two categories determined the final score, which was the sole focus of our analysis. The NI-RADS ranges from 1 to 4, with increasing values correlating to a higher likelihood of cancer recurrence. Given the study design, a category of 0 (indicative of a new baseline study without a comparable prior image) was not applicable [7].

For each MRI, we also assessed the individual features required for NI-RADS evaluation and classified them according to the risk of tumor recurrence or residual disease [15]: primary tumor size, classified as disappearance, reduction, stability, or increase; primary tumor signal on T2-weighted images, classified as appearance of hyperintensity or appearance of marked hypointensity, unchanged, or appearance of intermediate signal (“evil grey”); contrast enhancement of the primary tumor, classified as disappearance, diffuse linear, or focal; and lymph node size, classified as disappearance, decrease, stability, or increase. Furthermore, readers assessed diffusion restriction within the primary tumor on DWI sequences, categorizing it as decreased, unchanged, or increased.

**Fig. 1:**
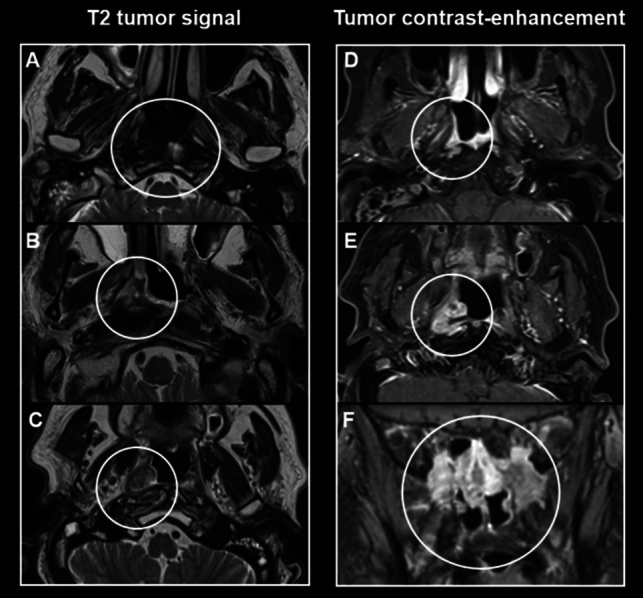
Examples of nasopharyngeal carcinoma features (white circles) after radiation therapy in T2-weighted images (**A**, **C**, **E**) and contrast-enhanced T1-weighted images (**B**, **D**, **F**). **B**, **D** and **C**, **E** from the same patients, respectively. **A** Hypointense prevertebral soft tissues, representing a scar. **B** Hyperintense soft tissue in the right pharyngeal recess, representing edema. **C** Intermediate hyperintense (“evil grey”) soft tissue in the right pharyngeal recess, representing tumor recurrence. **D** Thin linear mucosal enhancement in the right pharyngeal recess, representing inflammation. **E** Soft tissue with focal contrast enhancement, representing tumor recurrence. **F** Soft tissue with extensive contrast enhancement in the skull base foramina and perineural regions, representing tumor recurrence

See Fig. 1 as examples of main NPC features after radiation therapy in T2-weighted images and contrast-enhanced T1-weighted images.

### Statistical analysis

Interobserver reliability was assessed using Fleiss' kappa (*κ*) and percentage of agreement (POA) for all five readers, examining both overall NI-RADS scores and individual feature concordance (primary tumor size, signal on T2-weighted image, contrast enhancement, diffusion restriction, and lymph node size). Subgroup analyses were conducted to explore the influence of reader experience on NI-RADS reliability (comparing reader pairs A-B, A-C, and D-E, and grouping A, D, and E), and Cohen's *κ* was employed for two-reader comparisons. Furthermore, the agreement was examined separately for the first follow-up MRI and for the following scans. Kappa values were categorized to interpret agreement levels: < 0 (no agreement), 0.01–0.20 (slight), 0.21–0.40 (fair), 0.41–0.60 (moderate), 0.61–0.80 (substantial), and 0.81–0.99 (almost perfect) [16].

Finally, to assess the relationship between expert-assigned NI-RADS categories (reader A) and patient outcomes, a Chi-square test was conducted.

## Results

A cohort of 30 patients was studied, resulting in a total of 97 MRIs per rater and 485 MRIs for interrater agreement analysis. The most frequently assigned NI-RADS category was 1. Stability was indicated mainly for the features primary tumor size, primary tumor signal on T2-weighted, and lymph node size. In most cases, a decreased primary tumor contrast enhancement and a decreased in primary tumor diffusion restriction were indicated. Table 1 summarizes the distribution of data included and assigned in the study.

**Table 1:** Distribution of data included and assigned in the study

Variables	Values
Number of patients (male/female)	30 (25/5)
Mean age in years ± SD (range)	66 ± 13 (35–91)
Patients treated with chemotherapy concomitant with radiation therapy	2/30 (7%)
Patient outcomes within 2 years of the last follow-up (percentages):	
Disease-free status	25/30 (83%)
Disease recurrence or residual disease	5/30 (17%)
Mean maximum tumor size at diagnosis in millimeters ± SD (range)	33 ± 9 (14–58)
Number of patients based on MRI availability in follow-ups (percentages):	
First follow-up	26/30 (87%)
Second follow-up	27/30 (90%)
Third follow-up	24/30 (80%)
Fourth follow-up	20/30 (67%)
Mean time in days ± SD (range) between:	
Diagnosis and first follow-up	199 ± 118 (56–575)
Second and first follow-up	289 ± 160 (49–574)
Third and second follow-up	319 ± 216 (54–764)
Fourth and third follow-up	392 ± 195 (98–707)
Number of datasets per reader (total number of datasets compared between five readers):	
MRI	97 (485)
NI-RADS	97 (485)
Primary tumor size	97 (485)
Primary tumor T2-weighted signal	97 (485)
Primary tumor contrast enhancement	95 (475)
Primary tumor diffusion restriction on DWI	84 (420)
Lymph node size	97 (485)
Relative frequency of the variables assigned by the five readers (percentages)	
NI-RADS categories:	
1	392/485 (81%)
2	74/485 (15%)
3	19/485 (4%)
Primary tumor size:	
Disappearance	137/485 (28%)
Reduction	73/485 (15%)
Stability	256/485 (53%)
Increase	19/485 (4%)
Primary tumor T2-weighted signal:	
Hyperintensity or marked hypointensity	146/485 (30%)
Stability	322/485 (66%)
Intermediate intensity	17/485 (4%)
Primary tumor contrast enhancement:	
Disappearance	368/475 (77%)
Diffuse linear	94/475 (20%)
Focal	13/475 (3%)
Primary tumor diffusion restriction on DWI:	
Decreased	241/420 (57%)
Unchanged	27/420 (7%)
Increased	152/420 (36%)
Lymph node size:	
Disappearance	106/485 (22%)
Decrease	66/485 (14%)
Stability	311/485 (63%)
Increase	2/485 (1%)

SD, standard deviation; DWI, diffusion-weighted imaging; MRI, magnetic resonance imaging; NI-RADS, Neck Imaging Reporting and Data System

**Fig. 2:**
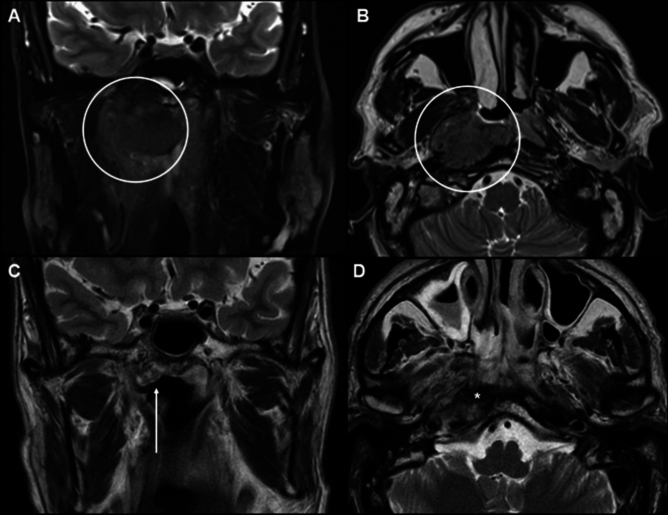
An example of low agreement in T2-weighted imaging evaluation of nasopharyngeal carcinoma (NPC) after radiotherapy. **A**, **B** are images before treatment, showing a large NPC with intermediate intensity on the right side of the nasopharynx. **C**, **D** are images after treatment, showing resolution of NPC, a hypointense post-radiation scar (asterisk) and a hyperintense soft tissue on the periphery of the scar representing edema (arrow), as confirmed in the subsequent follow-ups. In this case, one expert radiologist and two radiology residents classified the NPC size as reduced, while one expert radiologist and one general radiologist classified the NPC size as disappeared. One radiology resident classified the NPC signal on T2-weighted images as stable, while the other readers classified it as markedly hypointense. The final NI-RADS assigned was 1 for three readers (expert radiologist, general radiologist, and a radiology resident) and 2 for two readers (expert radiologist and a radiology resident)

All the radiologists agreed on NI-RADS assignment in 60/97 (62%) cases, four radiologists agreed in 20/97 (21%) cases, three radiologists agreed in 16/97 (16%), and two radiologists agreed in 1/97 (1%) cases. Complete disagreement among the readers never occurred. Interreader agreement values for all MRIs, for first follow-up MRIs only, and for all MRIs excluding the first follow-up are presented in Tables 2, 3, and 4, respectively. Additional tables displaying the interobserver agreement analyzed separately for the second, third, and fourth follow-ups are available in the supplementary information. Interreader agreement between all readers was moderate for NI-RADS (*κ* = 0.41, POA = 81%) and primary tumor size assessment (*κ* = 0.43, POA = 65%), while it was substantial for primary tumor signal on T2-weighted images (*κ* = 0.68, POA = 85%), diffusion restriction (*κ* = 0.70, POA = 84%), and lymph node size assessment (*κ* = 0.68, POA = 83%). Primary tumor contrast enhancement had the lowest *κ* value (*κ* = 0.27, POA = 74%). Specifically, subgroup analysis showed slight agreement for primary tumor contrast enhancement between the two radiology residents (*κ* = 0.20, POA = 63%) and fair agreement between the head and neck expert radiologist and the two radiology residents (*κ* = 0.21, POA = 69%). Interrater agreement at the first follow-up between all readers was fair for NI-RADS assignment (*κ* = 0.21, POA = 76%). The low reliability for primary tumor contrast enhancement evaluation between the two radiology residents (*κ* = 0.10, POA = 42%) and between the head and neck expert radiologist and the two radiology residents (*κ* = 0.02, POA = 31%) was confirmed. In addition, the first follow-up showed low agreement for the primary tumor size assessment between the two radiology residents (*κ* = 0.10, POA = 54%) and between the head and neck expert radiologist and the two radiology residents (*κ* = 0.17, POA = 38%). Conversely, during the initial follow-up, there was a high level of agreement in the analysis of DWI sequences among both the less experienced radiologists (*κ* = 0.65, POA = 95%) and between the less experienced radiologists and the more experienced one (*κ* = 0.79, POA = 96%). Overall, interrater agreement at second, third, and fourth follow-up between all readers was moderate for NI-RADS assignment (*κ* = 0.43, POA = 93%). Moreover, reliability for primary tumor contrast enhancement and size assessment was higher in subsequent follow-ups compared to the first follow-up, both between the two residents and between the expert radiologist and the residents. See Fig. 2 as an example of low agreement between readers in the evaluation of a NPC in the first follow-up after radiation therapy.

**Table 2:** Interrater agreement. Fleiss’ kappa is used for five- and three-reader reliability and Cohen’s kappa is used for two-reader reliability

	Variables	Kappa	Level of agreement according to kappa	Percentage of agreement (%)
Five readers (A, B, C, D, E)	NI-RADS	0.41 [CI 95%: 0.12, 0.70]	Moderate	81
	Primary tumor			
	Size	0.43 [CI 95%: 0.27, 0.59]	Moderate	65
	T2w signal	0.68 [CI 95%: 0.47, 0.89]	Substantial	85
	Diffusion restriction	0.70 [CI 95%: 0.50, 0.90]	Substantial	84
	Contrast enhancement	0.27 [CI 95%: 0.003, 0.54]	Fair	74
	Lymph node size	0.68 [CI 95%: 0.49, 0.87]	Substantial	83
Two readers (A, B)	NI-RADS	0.50 [CI 95%: 0.22, 0.74]	Moderate	87
	Primary tumor			
	Size	0.53 [CI 95%: 0.38, 0.69]	Moderate	73
	T2w signal	0.75 [CI 95%: 0.61, 0.89]	Substantial	89
	Diffusion restriction	0.61 [CI 95%: 0.45, 0.77]	Substantial	79
	Contrast enhancement	0.63 [CI 95%: 0.41, 0.80]	Substantial	88
	Lymph node size	0.79 [CI 95%: 0.63, 0.89]	Substantial	88
Two readers (A, C)	NI-RADS	0.43 [CI 95%: 0.19, 0.67]	Moderate	81
	Primary tumor			
	Size	0.53 [CI 95%: 0.38, 0.68]	Moderate	72
	T2w signal	0.67 [CI 95%: 0.51, 0.80]	Substantial	84
	Diffusion restriction	0.65 [CI 95%: 0.49, 0.80]	Substantial	81
	Contrast enhancement	0.38 [CI 95%: 0.14, 0.60]	Fair	81
	Lymph node size	0.76 [CI 95%: 0.64, 0.89]	Substantial	88
Two readers (D, E)	NI-RADS	0.47 [CI 95%: 0.24, 0.69]	Moderate	81
	Primary tumor			
	Size	0.41 [CI 95%: 0.26, 0.56]	Moderate	62
	T2w signal	0.64 [CI 95%: 0.48, 0.79]	Substantial	84
	Diffusion restriction	0.86 [CI 95%: 0.75, 0.97]	Almost perfect	93
	Contrast enhancement	0.20 [CI 95%: 0.06, 0.36]	Slight	63
	Lymph node size	0.75 [CI 95%: 0.62, 0.88]	Substantial	87
Three readers (A, D, E)	NI-RADS	0.49 [CI 95%: 0.12, 0.78]	Moderate	84
	Primary tumor			
	Size	0.44 [CI 95%: 0.28, 0.60]	Moderate	66
	T2w signal	0.73 [CI 95%: 0.51, 0.95]	Substantial	88
	Diffusion restriction	0.86 [CI 95%: 0.64, 1]	Almost perfect	93
	Contrast enhancement	0.21 [CI 95%: − 0.04, 0.46]	Fair	69
	Lymph node size	0.73 [CI 95%: 0.54, 0.92]	Substantial	86

Percentage of agreement is the total number of cases in which all readers agree, divided by the total number of observations. A and B: expert head and neck radiologists; C: general radiologist; D and E: radiology residents; NI-RADS, Neck Imaging Reporting and Data System; T2w, T2-weighted; CI, confidence interval

**Table 3:** Interrater agreement at first follow-up. Fleiss’ kappa is used for five- and three-reader reliability and Cohen’s kappa is used for two-reader reliability

	Variables	Kappa	Level of agreement according to kappa	Percentage of agreement (%)
Five readers (A, B, C, D, E)	NI-RADS	0.21 [CI 95%: 0.05, 0.37]	Fair	76
	Primary tumor			
	Size	0.24 [CI 95%: 0.09, 0.40]	Fair	78
	T2w signal	0.05 [CI 95%: − 0.38, 0.49]	Slight	93
	Diffusion restriction	0.51 [CI 95%: 0.24, 0.77]	Moderate	95
	Contrast enhancement	0.14 [CI 95%: − 0.05, 0.32]	Slight	78
	Lymph node size	0.47 [CI 95%: 0.35, 0.59]	Moderate	85
Two readers (A, B)	NI-RADS	0.21 [CI 95%: − 0.21, 0.63]	Fair	69
	Primary tumor			
	Size	0.36 [CI 95%: − 0.09, 0.80]	Fair	77
	T2w signal	0	-	88
	Diffusion restriction	0.53 [CI 95%: 0.05, 1]	Moderate	88
	Contrast enhancement	0.60 [CI 95%: 0.11, 1]	Moderate	88
	Lymph node size	0.44 [CI 95%: − 0.02, 0.90]	Moderate	73
Two readers (A, C)	NI-RADS	0.43 [CI 95%: − 0.03, 0.89]	Moderate	77
	Primary tumor			
	Size	0.43 [CI 95%: − 0.03, 0.89]	Moderate	77
	T2w signal	0	-	88
	Diffusion restriction	0.47 [CI 95%: 0.003, 0.94]	Moderate	92
	Contrast enhancement	0.02 [CI 95%: − 0.37, 0.41]	Slight	69
	Lymph node size	0.54 [CI 95%: 0.06, 1]	Moderate	77
Two readers (D, E)	NI-RADS	0.10 [CI 95%: − 0.30, 0.51]	Slight	54
	Primary tumor			
	Size	0.10 [CI 95%: − 0.30, 0.51]	Slight	54
	T2w signal	0	-	88
	Diffusion restriction	0.65 [CI 95%: 0.15, 1]	Substantial	96
	Contrast enhancement	0.10 [CI 95%: − 0.30, 0.50]	Slight	42
	Lymph node size	0.57 [CI 95%: 0.09, 1]	Moderate	77
Three readers (A, D, E)	NI-RADS	0.17 [CI 95%: − 0.12, 0.46]	Slight	38
	Primary tumor			
	Size	0.17 [CI 95%: − 0.12, 0.46]	Slight	38
	T2w signal	0	-	88
	Diffusion restriction	0.79 [CI 95%: 0.42, 1]	Substantial	96
	Contrast enhancement	0.02 [CI 95%: − 0.25, 0.30]	Slight	31
	Lymph node size	0.61 [CI 95%: 0.26, 0.95]	Substantial	69

Percentage of agreement is the total number of cases in which all readers agree, divided by the total number of observations. A and B: expert head and neck radiologists; C: general radiologist; D and E: radiology residents; NI-RADS, Neck Imaging Reporting and Data System; T2w, T2-weighted; CI, confidence interval

**Table 4:** Interrater agreement at second, third, and fourth follow-up. Fleiss’ kappa is used for five- and three-reader reliability and Cohen’s kappa is used for two-reader reliability

	Variables	Kappa	Level of agreement according to kappa	Percentage of agreement (%)
Five readers (A, B, C, D, E)	NI-RADS	0.43 [CI 95%: 0.39, 0.48]	Moderate	93
	Primary tumor			
	Size	0.22 [CI 95%: 0.19, 0.25]	Fair	78
	T2w signal	0.23 [CI 95%: 0.20, 0.26]	Fair	91
	Diffusion restriction	0.42 [CI 95%: 0.37, 0.47]	Moderate	89
	Contrast enhancement	0.33 [CI 95%: 0.28, 0.38]	Fair	87
	Lymph node size	0.45 [CI 95%: 0.42, 0.48]	Moderate	93
Two readers (A, B)	NI-RADS	0.64 [CI 95%: 0.58, 0.70]	Substantial	93
	Primary tumor			
	Size	0.20 [CI 95%: 0.09, 0.30]	Slight	72
	T2w signal	0.29 [CI 95%: 0.22, 0.37]	Fair	89
	Diffusion restriction	0.34 [CI 95%: 0.21, 0.44]	Fair	74
	Contrast enhancement	0.64 [CI 95%: 0.56, 0.71]	Substantial	88
	Lymph node size	0.67 [CI 95%: 0.61, 0.73]	Substantial	93
Two readers (A, C)	NI-RADS	0.40 [CI 95%: 0.31, 0.49]	Fair	83
	Primary tumor			
	Size	0.26 [CI 95%: 0.15, 0.36]	Fair	70
	T2w signal	0.19 [CI 95%: 0.10, 0.28]	Slight	82
	Diffusion restriction	0.32 [CI 95%: 0.21, 0.43]	Fair	76
	Contrast enhancement	0.52 [CI 95%: 0.44, 0.61]	Moderate	86
	Lymph node size	0.61 [CI 95%: 0.54, 0.67]	Substantial	92
Two readers (D, E)	NI-RADS	0.43 [CI 95%: 0.36, 0.49]	Moderate	92
	Primary tumor			
	Size	0.30 [CI 95%: 0.18, 0.41]	Fair	65
	T2w signal	0.11 [CI 95%: 0.02, 0.20]	Slight	82
	Diffusion restriction	0.65 [CI 95%: 0.58, 0.72]	Substantial	91
	Contrast enhancement	0.22 [CI 95%: 0.11, 0.32]	Fair	71
	Lymph node size	0.62 [CI 95%: 0.55, 0.69]	Substantial	90
Three readers (A, D, E)	NI-RADS	0.59 [CI 95%: 0.51, 0.66]	Moderate	90
	Primary tumor			
	Size	0.24 [CI 95%: 0.11, 0.38]	Fair	53
	T2w signal	0.19 [CI 95%: 0.08, 0.30]	Slight	79
	Diffusion restriction	0.63 [CI 95%: 0.52, 0.73]	Substantial	86
	Contrast enhancement	0.29 [CI 95%: 0.16, 0.42]	Fair	65
	Lymph node size	0.50 [CI 95%: 0.40, 0.61]	Moderate	82

Percentage of agreement is the total number of cases in which all readers agree, divided by the total number of observations. A and B: expert head and neck radiologists; C: general radiologist; D and E: radiology residents; NI-RADS, Neck Imaging Reporting and Data System; T2w, T2-weighted; CI, confidence interval

In patients with residual or recurrent disease, the interreader agreement of NI-RADS among the five observers, calculated by Fleiss' *κ*, was 0.41 [CI 95%: 0.39–0.42] with an overall agreement rate of 61%. In disease-free patients, the interreader agreement of NI-RADS among the five observers, calculated by Fleiss' *κ*, was 0.34 [CI 95%: 0.29–0.36] with an overall agreement rate of 65%.

Using NI-RADS categories of the expert radiologist as reference standard, the Chi-square test yielded a significant result (*p* < 0.001), indicating a strong association between NI-RADS and patient outcomes. Specifically, our contingency table revealed that all patients with a NI-RADS score of 1 were in remission, while all patients with a NI-RADS score of 3 experienced recurrence. Patients with a NI-RADS score of 2 had an equal distribution of remission and recurrence, although the sample size for this group was very small.

## Discussion

In our study, MRI NI-RADS showed a moderate interrater agreement among readers with different levels of experience. In particular, the features primary tumor signal on T2-weighted images, diffusion restriction, and lymph node size exhibited good reliability. Conversely, primary tumor size and contrast enhancement exhibited lower interreader agreement. The results were influenced by reader experience, as demonstrated by the fact that the subgroup analysis of less experienced radiologists showed lower agreement values compared to those with greater experience. Moreover, a separate sub-analysis was conducted for the first and subsequent post-treatment MRIs to determine the influence of early radiation therapy effects on MRI interpretation. This approach aligns with the guidelines provided by the NI-RADS, which distinguishes the initial post-treatment scan from subsequent examinations [7]. In our cohort, the reliability of the first follow-up examinations was lower than that of subsequent controls, especially in the subgroup including less experienced radiologists. These data suggest that the first post-radiotherapy MRI is more challenging to evaluate for young radiologists, leading to a more cautious interpretation in indicating residual tumor rather than a completely disappeared one or in differentiating post-irradiation contrast enhancement from that suspicious for residual disease. This ultimately influenced the choice of NI-RADS, overestimating a score of 2 when a score of 1 could have been appropriate. Furthermore, DWI emerged as a consistent parameter even for younger radiologists. Despite not being strictly included among the criteria necessary for NI-RADS calculation, DWI has already demonstrated an improvement in the diagnostic accuracy of the score [17]. Therefore, on the one hand, our data suggest the need for greater attention and educational efforts toward younger radiologists to differentiate normal post-radiotherapy findings from a tumor recurrence. On the other hand, our results underline the importance of multiparametric MRI assessment and, in particular, of DWI, which has shown good interobserver agreement. To assess the interreader agreement, we employed both the POA and the *κ* statistic as reported in Tables 2, 3, and 4. However, comparison of these two measures revealed discrepancies in some cases. For example, primary tumor contrast enhancement among all readers yielded a low *κ* (0.27) and a high POA (74%), or primary tumor T2-weighted signal at the first follow-up showed a *κ* of 0.05 and a POA of 93%. This disparity can arise when a majority of subjects fall into the same category. Such instances inflate the probability of chance agreement, consequently reducing the *κ* value [18, 19].

To our knowledge, no other research has specifically examined interobserver agreement in MRI NI-RADS assessment among radiologists with varying expertise in NPC surveillance. Consequently, direct comparison with existing literature is challenging, and variations exist, likely due to differences in study populations, imaging techniques, and readers’ experience. Furthermore, recognizing the potential learning curve associated with new templates or structured reporting systems is crucial. It is plausible that interobserver agreement may enhance with the widespread integration of this scoring system into routine clinical practice.

Elsholtz et al. showed results similar to ours in a cohort of 104 patients with diverse head and neck cancers, including nine NPC, who underwent surveillance contrast-enhanced MRI. Three experienced head and neck radiologists independently assessed these images. Moderate interobserver agreement was found for NI-RADS categorization of the primary tumor site (Fleiss’ *κ* = 0.53), while substantial agreement was observed for neck nodal assessment (Fleiss’ *κ* = 0.67). Excellent consistency was noted for DWI of the primary tumor (Fleiss’ *κ* = 0.83) [14]. Abdelaziz et al. examined a cohort of treated head and neck squamous cell carcinomas, including ten NPC, and reported near-perfect interobserver agreement among two expert head and neck radiologists when excluding primary neck lesion enhancement using CT or MRI (*κ* = 0.83, POA = 96.4%). Substantial agreement was observed for identifying discrete nodular or diffuse linear mucosal enhancement (*κ* = 0.73 and 0.71, respectively). A focused analysis of MRI data alone demonstrated substantial concordance among observers in assessing the primary tumor site (*κ* = 0.78, POA = 85%). Furthermore, nearly perfect agreement was observed for lymph node evaluation (*κ* = 0.85, POA = 91%) [13]. A separate study examining 50 oral squamous cell carcinoma patients after surgery, using mainly CT, reported moderate interobserver agreement among four expert radiologists in assessing the primary site and substantial agreement for neck nodal evaluation. Combined assessment of both sites yielded strong agreement [9].

Our results align with the established link between NI-RADS score and patient outcomes [20]. Specifically, we found a strong association between a NI-RADS score of 1 and remission, while a score of 3 was strongly linked to recurrence. However, due to the relatively small sample size, the relationship between a NI-RADS score of 2 and patient outcomes cannot be established in our study.

Several limitations inherent to this study warrant consideration. The retrospective design hindered comprehensive access to patient clinical data. The relatively small patient cohort might be viewed as a study limitation; however, given NI-RADS' role in surveillance rather than diagnosis, the emphasis should be on the substantial number of follow-up MRI examinations (*n* = 97) analyzed longitudinally, rather than the patient count (*n* = 30). The relatively small patient cohort, especially those with residual or recurrent disease, limits our ability to establish a definitive correlation between NI-RADS and histopathology. Similarly, the small number of patients precludes evaluating the potential influence of specific patient-related factors on the NI-RADS scoring. Nonetheless, these were not the primary focus of the current investigation. Furthermore, the analysis encompassed MRI data acquired across multiple scanners. While this reflects real-world radiology practice, it introduces potential variability in image acquisition. Consequently, standardizing imaging protocols was challenging. In a small subset of cases, post-contrast images or diffusion-weighted imaging were absent due to the lack of standardized head and neck imaging protocols in earlier examinations. Moreover, the study encompassed a broad temporal range. Given the low prevalence of the patient population with NPC in Italy, a lengthy study period was essential to achieving adequate patient enrollment.

Future studies could assess interreader agreement of NI-RADS in MRI among radiologists with varying experience in other types of head and neck cancers. Also, larger sample sizes will be necessary to evaluate the potential impact of individual patient features (e.g., the use of treatment modalities other than radiotherapy alone or the tumor size at diagnosis) on reliability.

## Conclusion

MRI NI-RADS has a moderate interrater agreement for the surveillance of NPC patients after radiation therapy among readers with different levels of experience. Reliability has the potential to increase, particularly by enhancing focus from less experienced radiologists on the assessment of primary tumor contrast enhancement and size and by adding DWI evaluation in the first examination performed after radiation therapy.

## Supplementary Information

Below is the link to the electronic supplementary material.

Supplementary file1 (PDF 160 kb)
