# Supplementary material for: Interrater reliability of MRI Neck Imaging Reporting and Data System (NI-RADS) in the follow-up of nasopharyngeal carcinoma after radiation therapy
Source: Radiol Med. 2025 Apr 1;130(6):854–64. doi: 10.1007/s11547-025-01982-4 (PMC12185612; doi:10.1007/s11547-025-01982-4)
Supplement: Supplementary file 1 — Supplementary file1 (PDF 160 kb) [file 11547_2025_1982_MOESM1_ESM.pdf]

Table 5 Interrater agreement at second follow up. Fleiss' kappa is used for 5 and 3 readers reliability and Cohen's kappa is used for 2 readers reliability. Percentage of agreement is the total number of cases in which all readers agree, divided by the total number of observations. A and B: expert head and neck radiologists; C: general radiologist; D and E: radiology residents; NI-RADS, Neck Imaging Reporting and Data System; T2w, T2-weighted; CI, confidence interval.

	Variables	Kappa	Level of agreement according to kappa	Percentage of agreement
5 readers (A, B, C, D, E)	NI-RADS	0.46 [CI 95%: 0.21, 0.71]	Moderate	87%
	Primary tumor			
	Size	0.16 [CI 95%: -0.09, 0.42]	Slight	51%
	T2w signal	0.32 [CI 95%: 0.06, 0.58]	Fair	82%
	Diffusion restriction	0.54 [CI 95%: 0.35, 0.73]	Moderate	80%
	Contrast-enhancement	0.19 [CI 95%: -0.02, 0.40]	Slight	72%
	Lymph node size	0.45 [CI 95%: 0.35, 0.71]	Moderate	83%
2 readers (A, B)	NI-RADS	0.72 [CI 95%: 0.58, 0.87]	Substantial	93%
	Primary tumor			
	Size	0.22 [CI 95%: 0.04, 0.41]	Fair	63%
	T2w signal	0.53 [CI 95%: 0.35, 0.70]	Moderate	89%
	Diffusion restriction	0.40 [CI 95%: 0.21, 0.58]	Fair	74%
	Contrast-enhancement	0.75 [CI 95%: 0.63, 0.88]	Substantial	92%
	Lymph node size	0.70 [CI 95%: 0.56, 0.83]	Substantial	89%
2 readers (A, C)	NI-RADS	0.59 [CI 95%: 0.42, 0.75]	Moderate	89%
	Primary tumor			
	Size	0.21 [CI 95%: 0.03, 0.40]	Fair	59%
	T2w signal	0.17 [CI 95%: -0.02, 0.36]	Slight	78%
	Diffusion restriction	0.44 [CI 95%: 0.26, 0.63]	Moderate	74%
	Contrast-enhancement	0.18 [CI 95%: -0.01, 0.37]	Slight	81%
	Lymph node size	0.78 [CI 95%: 0.66, 0.90]	Substantial	93%
2 readers (D, E)	NI-RADS	0.34 [CI 95%: 0.17, 0.54]	Fair	89%
	Primary tumor			
	Size	0.06 [CI 95%: -0.13, 0.24]	Slight	41%
	T2w signal	0.29 [CI 95%: 0.10, 0.48]	Slight	81%
	Diffusion restriction	0.78 [CI 95%: 0.65, 0.91]	Substantial	91%
	Contrast-enhancement	0.08 [CI 95%: -0.11, 0.28]	Slight	54%
	Lymph node size	0.70 [CI 95%: 0.57, 0.84]	Substantial	89%
3 readers (A, D, E)	NI-RADS	0.46 [CI 95%: 0.07, 0.84]	Moderate	85%
	Primary tumor			
	Size	0.10 [CI 95%: -0.29, 0.49]	Slight	22%
	T2w signal	0.29 [CI 95%: -0.11, 0.69]	Fair	74%
	Diffusion restriction	0.77 [CI 95%: 0.54, 0.99]	Substantial	87%
	Contrast-enhancement	0.10 [CI 95%: -0.23, 0.43]	Slight	50%
	Lymph node size	0.59 [CI 95%: 0.33, 0.86]	Moderate	78%

Table 6 Interrater agreement at third follow up. Fleiss' kappa is used for 5 and 3 readers reliability and Cohen's kappa is used for 2 readers reliability. Percentage of agreement is the total number of cases in which all readers agree, divided by the total number of observations. A and B: expert head and neck radiologists; C: general radiologist; D and E: radiology residents; NI-RADS, Neck Imaging Reporting and Data System; T2w, T2-weighted; CI, confidence interval.

	**Variables**	**Kappa**	**Level of agreement according to kappa**	**Percentage of agreement**
5 readers (A, B, C, D, E)	NI-RADS	0.41 [CI 95%: 0.14, 0.68]	Moderate	75%
	Primary tumor			
	Size	0.11 [CI 95%: -0.16, 0.38]	Slight	46%
	T2w signal	0.12 [CI 95%: -0.15, 0.39]	Slight	67%
	Diffusion restriction	0.45 [CI 95%: 0.23, 0.67]	Moderate	65%
	Contrast-enhancement	0.37 [CI 95%: 0.16, 0.58]	Fair	58%
	Lymph node size	0.20 [CI 95%: -0.07, 0.47]	Slight	75%
2 readers (A, B)	NI-RADS	0.35 [CI 95%: 0.15, 0.55]	Fair	88%
	Primary tumor			
	Size	0	-	71%
	T2w signal	0	-	88%
	Diffusion restriction	0.40 [CI 95%: 0.20, 0.60]	Fair	75%
	Contrast-enhancement	0.32 [CI 95%: 0.13, 0.51]	Fair	79%
	Lymph node size	0	-	92%
2 readers (A, C)	NI-RADS	0.29 [CI 95%: 0.09, 0.49]	Fair	79%
	Primary tumor			
	Size	0.21 [CI 95%: 0.01, 0.41]	Fair	75%
	T2w signal	0.36 [CI 95%: 0.16, 0.56]	Fair	87%
	Diffusion restriction	0.50 [CI 95%: 0.31, 0.69]	Moderate	85%
	Contrast-enhancement	0.73 [CI 95%: 0.60, 0.87]	Substantial	92%
	Lymph node size	0.47 [CI 95%: 0.28, 0.66]	Moderate	92%
2 readers (D, E)	NI-RADS	0.47 [CI 95%: 0.28, 0.66]	Moderate	92%
	Primary tumor			
	Size	0.42 [CI 95%: 0.22, 0.61]	Moderate	79%
	T2w signal	0	-	79%
	Diffusion restriction	0.46 [CI 95%: 0.26, 0.65]	Moderate	90%
	Contrast-enhancement	0.30 [CI 95%: 0.11, 0.49]	Fair	75%
	Lymph node size	0.64 [CI 95%: 0.47, 0.81]	Substantial	92%
3 readers (A, D, E)	NI-RADS	0.69 [CI 95%: 0.34, 1.03]	Substantial	92%
	Primary tumor			
	Size	0.13 [CI 95%: -0.28, 0.55]	Slight	67%
	T2w signal	0.11 [CI 95%: -0.31, 0.53]	Slight	79%
	Diffusion restriction	0.69 [CI 95%: 0.41, 0.96]	Substantial	90%
	Contrast-enhancement	0.43 [CI 95%: 0.11, 0.74]	Moderate	71%
	Lymph node size	0.31 [CI 95%: -0.11, 0.74]	Fair	79%

Table 7 Interrater agreement at fourth follow up. Fleiss' kappa is used for 5 and 3 readers reliability and Cohen's kappa is used for 2 readers reliability. Percentage of agreement is the total number of cases in which all readers agree, divided by the total number of observations. A and B: expert head and neck radiologists; C: general radiologist; D and E: radiology residents; NI-RADS, Neck Imaging Reporting and Data System; T2w, T2-weighted; CI, confidence interval.

	**Variables**	**Kappa**	**Level of agreement according to kappa**	**Percentage of agreement**
5 readers (A, B, C, D, E)	NI-RADS	0.41 [CI 95%: 0.11, 0.70]	Moderate	75%
	Primary tumor			
	Size	0.30 [CI 95%: -0.002, 0.60]	Fair	55%
	T2w signal	0.14 [CI 95%: -0.15, 0.44]	Slight	70%
	Diffusion restriction	0.34 [CI 95%: 0.11, 0.58]	Fair	58%
	Contrast-enhancement	0.45 [CI 95%: 0.23, 0.67]	Moderate	65%
	Lymph node size	0.27 [CI 95%: 0.03, 0.50]	Fair	85%
2 readers (A, B)	NI-RADS	1	Perfect agreement	100%
	Primary tumor			
	Size	0.37 [CI 95%: 0.15, 0.58]	Fair	85%
	T2w signal	0	-	90%
	Diffusion restriction	0.28 [CI 95%: 0.06, 0.50]	Fair	74%
	Contrast-enhancement	0.85 [CI 95%: 0.73, 0.96]	Almost perfect	95%
	Lymph node size	1	Perfect agreement	100%
2 readers (A, C)	NI-RADS	0.30 [CI 95%: 0.08, 0.51]	Fair	80%
	Primary tumor			
	Size	0.30 [CI 95%: 0.08, 0.51]	Fair	80%
	T2w signal	0	-	80%
	Diffusion restriction	0.16 [CI 95%: 0.31, 0.69]	Slight	68%
	Contrast-enhancement	0.59 [CI 95%: 0.41, 0.77]	Moderate	85%
	Lymph node size	0	-	90%
2 readers (D, E)	NI-RADS	0.49 [CI 95%: 0.28, 0.69]	Moderate	95%
	Primary tumor			
	Size	0.27 [CI 95%: 0.05, 0.48]	Fair	80%
	T2w signal	0	-	85%
	Diffusion restriction	0.62 [CI 95%: 0.44, 0.80]	Substantial	89%
	Contrast-enhancement	0.35 [CI 95%: 0.14, 0.55]	Fair	85%
	Lymph node size	0	-	90%
3 readers (A, D, E)	NI-RADS	0.65 [CI 95%: 0.26, 1.04]	Substantial	95%
	Primary tumor			
	Size	0.38 [CI 95%: -0.08, 0.84]	Fair	80%
	T2w signal	0	-	85%
	Diffusion restriction	0.44 [CI 95%: 0.08, 0.79]	Moderate	79%
	Contrast-enhancement	0.36 [CI 95%: 0.003, 0.71]	Fair	75%
	Lymph node size	0.30 [CI 95%: -0.06, 0.66]	Fair	90%
